# Pupillary Response to Cognitive Demand in Parkinson’s Disease: A Pilot Study

**DOI:** 10.3389/fnagi.2018.00090

**Published:** 2018-04-10

**Authors:** Melike Kahya, Sanghee Moon, Kelly E. Lyons, Rajesh Pahwa, Abiodun E. Akinwuntan, Hannes Devos

**Affiliations:** ^1^Laboratory for Advanced Rehabilitation Research in Simulation, Department of Physical Therapy and Rehabilitation Science, School of Health Professions, University of Kansas Medical Center, Kansas City, KS, United States; ^2^Department of Neurology, School of Medicine, University of Kansas Medical Center, Kansas City, KS, United States; ^3^Office of the Dean, School of Health Professions, University of Kansas Medical Center, Kansas City, KS, United States

**Keywords:** Parkinson’s disease, pupillary response, cognitive demand, non-demented, working memory

## Abstract

Previous studies have shown that pupillary response, a physiological measure of cognitive workload, reflects cognitive demand in healthy younger and older adults. However, the relationship between cognitive workload and cognitive demand in Parkinson’s disease (PD) remains unclear. The aim of this pilot study was to examine the pupillary response to cognitive demand in a letter-number sequencing (LNS) task between 16 non-demented individuals with PD (age, median (Q1–Q3): 68 (62–72); 10 males) and 10 control participants (age: 63 (59–67); 2 males), matched for age, education, and Montreal Cognitive Assessment (MOCA) scores. A mixed model analysis was employed to investigate cognitive workload changes as a result of incremental cognitive demand for both groups. As expected, no differences were found in cognitive scores on the LNS between groups. Cognitive workload, exemplified by greater pupil dilation, increased with incremental cognitive demand in both groups (*p* = 0.003). No significant between-group (*p* = 0.23) or interaction effects were found (*p* = 0.45). In addition, individuals who achieved to complete the task at higher letter-number (LN) load responded differently to increased cognitive demand compared with those who completed at lower LN load (*p* < 0.001), regardless of disease status. Overall, the findings indicated that pupillary response reflects incremental cognitive demand in non-demented people with PD and healthy controls. Further research is needed to investigate the pupillary response to incremental cognitive demand of PD patients with dementia compared to non-demented PD and healthy controls.

**Highlights**

- Pupillary response reflects cognitive demand in both non-demented people with PD and healthy controls
- Although not significant due to insufficient power, non-demented individuals with PD had increased cognitive workload compared to the healthy controls throughout the testing
- Pupillary response may be a valid measure of cognitive demand in non-demented individuals with PD
- In future, pupillary response might be used to detect cognitive impairment in individuals with PD

## Introduction

Parkinson’s disease (PD) is the second most prevalent neurodegenerative condition, characterized by motor and non-motor symptoms (Mhyre et al., 2012). Non-motor symptoms such as cognitive impairment, depression and fatigue are common in PD. These non-motor symptoms may reduce quality of life, perhaps even more so than motor symptoms (Cahn et al., 1998; Weintraub et al., 2004). Cognitive impairment can present in up to 25% of newly diagnosed people with PD (Pedersen et al., 2013). These mild cognitive impairment may eventually progress to dementia (Pedersen et al., 2013). Up to 46% of people with PD will develop dementia 10 years after the diagnosis, and up to 80% will develop dementia 20 years following diagnosis (Williams-Gray et al., 2013). Accurate detection of cognitive impairment is a critical step towards providing targeted diagnosis and treatment for cognitive impairment in PD (Espay et al., 2016).

Impairment in working memory is one of the first symptoms of cognitive dysfunction in PD, eventually resulting in diminished performance on activities of daily living and management of medication (Costa et al., 2015; Fallon et al., 2017). Working memory refers to the concurrent storage and information processing during dynamic cognitive activity (Bublak et al., 2002). This cognitive domain is especially necessary to manage increased cognitive demand when task-relevant information gradually increases (Bublak et al., 2002). Degeneration of dopaminergic cells in the substantia nigra are considered the main cause of impairments in working memory (Lewis et al., 2005). The loss of dopaminergic cells in the substantia nigra results in reduced activity in the ventro-lateral and dorso-lateral prefrontal cortices while performing a working memory task in PD (Lewis et al., 2003a,b). Deficits in noradrenergic pathways also contribute to working memory impairments in PD (Kehagia et al., 2010). To date, the cognitive workload exhibited in real-time during tasks of working memory in PD is not clear.

Advances in neurophysiological technology enable us to measure cognitive workload during cognitive testing in real-time (Kahneman, 1973; Ranchet et al., 2017a). Based on Kahneman’s attention theory, cognitive workload is defined as the mental effort that is needed to execute a task (Kahneman, 1973). The ability to perform well on a cognitive task depends on the available cognitive resources (Kahneman, 1973). When the cognitive demand is less than the available cognitive resources, the task will be executed accurately (Kahneman, 1973). The pupillary response is a neurophysiological measurement that has been shown to accurately measure cognitive workload in healthy younger and older individuals (Beatty, 1982; Marshall, 2007; Allard et al., 2010; Piquado et al., 2010). The locus coeruleus —a small nucleus in the brainstem—has a significant role in pupil response due to increased cognitive activity in the brain (Eckstein et al., 2017). In later stages of PD, noradrenergic and dopaminergic neuronal degeneration in the locus coeruleus could be associated with atypical, perhaps increased pupillary response during increased cognitive activity (Wang et al., 2016).

To our knowledge, the relationship between pupillary response and cognitive demand during a working memory task with incremental difficulty has not been examined in non-demented people with PD. The pupils have been shown to be sensitive to incremental increases in cognitive demand in people with mild cognitive impairment and in individuals with dementia (Elman et al., 2017; Granholm et al., 2017). Furthermore, it has been shown that individuals who performed high on the digit-span task exhibited lesser pupil dilation compared to the individuals who performed poorly on the same task (Granholm et al., 2017). This might suggest that pupillary response differentiates between high performers and low performers on the cognitive task. However, it is not known how PD pathophysiology affects pupillary response between high performers and low performers.

Demonstrating the validity of the pupillary response to cognitive demand might help to determine more sensitive measures of cognitive dysfunction in PD. Therefore, the purpose of this study was to investigate the relationship between pupillary response and cognitive demand in non-demented people with PD while performing a working memory test. The primary hypothesis of this study was that cognitive workload measured by pupillary response would reflect cognitive demand in non-demented people with PD and healthy controls. An exploratory aim was to investigate the pattern of cognitive workload as a result of task difficulty in non-demented people with PD and healthy controls who performed well and those who performed poorly on the task.

## Materials and Methods

### Participants

Non-demented participants with PD were recruited from the Parkinson’s Disease and Movement Disorder Center at the University of Kansas Medical Center (KUMC) between January and April 2017. Diagnosis of idiopathic PD was based on the UK Parkinson’s Disease Brain Bank Clinical Diagnostic Criteria (Hughes et al., 1992). Healthy controls were spouses of the participants with PD or recruited from the community through word-of-mouth. The study participation criteria were designed to exclude people with PD with cognitive impairment. Therefore, the inclusion criteria for both groups were: (1) Montreal Cognitive Assessment (MOCA) score >25; (2) non-demented individuals with PD whose scores lie within two standard deviations from normative values or from mean values of healthy controls on the cognitive battery to determine mild cognitive impairment (Litvan et al., 2012); and (3) voluntary consent. The exclusion criteria were: (1) diagnosis of mild cognitive impairment or dementia; (2) atypical parkinsonism; (3) secondary parkinsonism; (4) history of diagnosed but unresolved neurological, visual (e.g., glaucoma, cataract) or vestibular conditions unrelated to PD; (4) severe trunk and head dyskinesia or dystonia in the medication “ON” state; (5) blepharospasm; (6) deep brain stimulation; and (7) unpredictable motor fluctuations. The study was approved by the Human Subjects Committee at the University of Kansas Medical Center (KUMC), which complies with the Declaration of Helsinki (approval number 12490). Institutionally approved written consent was obtained before enrolment from all participants.

### Protocol

Participants completed demographic and medical information questionnaires regarding their disease symptoms, medical history, and medication use from which levodopa equivalent daily (LED) dose was calculated (Deuschl et al., 2006). Participants were administered the MOCA, which is a brief cognitive screening tool that evaluates eight different cognitive domains including executive function, attention, learning, and memory (Nasreddine et al., 2005). In addition, a battery of cognitive tests recommended by the Movement Disorder Society (Litvan et al., 2012) was administered to rule out participants with cognitive impairment (Table 1). Participants with PD were administered the Movement Disorder Society-Unified Parkinson’s Disease Rating Scale (MDS-UPDRS; Goetz et al., 2008) Part II (motor experiences of daily living) and Part III (motor examination), and Hoehn and Yahr (H&Y) Scale (Hoehn and Yahr, 1967). To control for a possible effect of autonomic dysfunction on pupillary response, subjects with PD completed the Scales for Outcomes in PD—Autonomic Dysfunction (SCOPA-AUT; Visser et al., 2004). SCOPA-AUT includes 25 items assessing the following regions: gastrointestinal (seven items), urinary (six items), cardiovascular (three items), thermoregulatory (four items), pupillomotor (one item), and sexual (six items for men and six items for women) dysfunction. All assessments were conducted in the medication “ON” state, approximately 45 min after medication intake.

**Table 1:** Descriptive statistics of patient demographic, psychometric and clinical characteristics.

Variable	PD (*n* = 16)	Control (*n* = 10)	Test	*p*-value
*Demographic characteristics*				
Age, years	68 (62–72)	63 (59–67)	110.50 (W)	0.20
Sex, male, *n*	10 (63)	2 (20)	4.47 (*χ*^2^)	0.03^a^
Education, years	16 (16–17)	18 (16–20)	191.00 (W)	0.20
*Psychometric characteristics*				
MOCA	28 (27–29)	28 (27–29)	202.50 (W)	0.48
*Attention and working memory*				
WAIS-IV LNS	9 (8–10)	10 (9–11)	196.50 (W)	0.31
Stroop Color-Word, *n*^b^	0 (0–0)	0 (0–0)	95.00 (W)	0.31
*Executive function*				
Tower of London, seconds	642 (483–758)	651 (549–874)	179.50 (W)	0.64
*Language*				
WAIS-IV Similarities	8 (8–10)	9 (8–11)	202.00 (W)	0.48
Boston Naming	15 (15–15)	15 (15–15)	206.00 (W)	0.62
CVLT-II Trial 1–5	43 (35–51)	52 (43–62)	175.50 (W)	0.03^a^
*Memory*				
WMS-IV Logical Memory I	9 (8–11)	12 (10–12)	169.00 (W)	0.01^a^
WMS-IV Logical Memory II	9 (7–11)	10 (9–12)	176.00 (W)	0.07
*Visuospatial function*				
Clock copy	9 (9–9)	10 (9–10)	177.50 (W)	0.34
Rey-Osterrieth Figure	31 (29–32)	30 (28–31)	73.00 (W)	0.63
*Clinical characteristics*				
Disease duration, *years*	6 (3–7)	NA		
UPDRS II	10 (7–15)	NA		
UPDRS III	30 (24–39)	NA		
H & Y	2 (2–2)	NA		
SCOPA-AUT	14 (9–14)	NA		
LED	750 (544–1037)	NA		

*^a^Statistically significant (p < 0.05). ^b^Number of incorrect answers. Values are expressed as median (Q1–Q3) or number (%). Abbreviation: *χ*^2^, chi-square test; CVLT-II, California Verbal Learning Test-II; H &Y, Hoehn and Yahr stage; LED, levodopa equivalent dose; LNS, letter-number sequencing test; NA, not applicable; MOCA, Montreal Cognitive Assessment; PD, Parkinson’s disease; SCOPA-AUT, Scales for Outcomes in Parkinson’s Disease-Autonomic questionnaire; UPDRS, Unified Parkinson Disease Rating Scale; W, Wilcoxon rank sum test; WAIS, Wechsler Adult Intelligence Scale; WMS, Wechsler Memory Scale*.

The letter-number sequencing (LNS) test of the Wechsler Adult Intelligence Scale-IV (WAIS-IV) was administered to assess incremental demand of working memory using an iPad-based application from Q-interactive (Pearson Inc., 2017; Wechsler, 2008). Participants were asked to recall a sequence of scrambled letters and numbers by first repeating the sequence of numbers in ascending order followed by the sequence of letters in alphabetical order. The test ended when the participant incorrectly recalled the sequence on three consecutive trials or achieved the maximum LN load. The maximum score (scaled) for this test is 19 points, which indicates well-functioning working memory. This test consists of a series of questions with increasing difficulty from two LN loads to eight LN loads, in which higher LN load indicates higher task difficulty. The test was administered by a trained research assistant in a single session.

### Cognitive Workload Assessment

**Figure 1:**
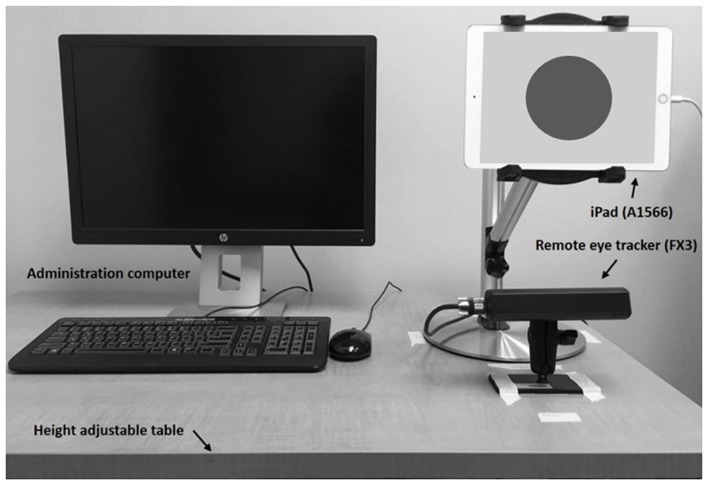
Experimental setup for the remote eye tracking system.

Participants were seated in front of the 9.7″ iPad Air 2 (A1566, Apple Inc., 2014) mounted on a stand at about eye height on a height-adjustable table (Figure 1). A remote eye tracker (FX3, SeeingMachines Inc.) was placed right underneath the iPad and recorded raw pupil size at 60 Hz while participants were performing cognitive tasks. This remote setup of the eye tracker did not require any actual physical contact, such as a chin rest or head-mounted camera, between the participant and the device. Although this setup allows pupil measurements without any distractions, participants were asked to focus on a red dot with a radius of 4.5 cm on the iPad screen during the tasks in order to minimize missing variables collected by the remote eye tracker using the EyeWorks™ Record software (EyeTracking Inc., 2011). Video and audio were recorded throughout the LNS task using the same software for analysis.

Pupillary responses were transformed into the Index of Cognitive Activity (ICA). ICA is the proportion of observations in a second that reflect significant mental effort, with signal smoothing to eliminate statistical noise and hardware anomalies. The ICA is based on the transformation of pupil diameter through signal processing algorithms of wavelet analysis. It focuses on the dilation reflex similar to task-evoked pupillary response, but also takes into account both the rapid constrictions of the pupil that result from increased light and the relatively slow dilations that result from accommodation to decreased light (Marshall, 2007). In the current study, ICA method was used to quantify cognitive workload during the cognitive testing. The EyeWorks™ Analyze software (EyeTracking Inc., 2011) was used to transform pupil dilation data from the right eye to the scaled ICA that ranges between 0 and 1. Although right eye and left eye ICA results were strongly correlated (*r* = 0.70, *p* < 0.001), right eye ICA data was shown interpretable results compared to the left eye ICA data which was also consistent with the previous literature (Gangeddula et al., 2017; Ranchet et al., 2017b). Recorded videos were analyzed to find start- and end-time points of each LN load. Then, scaled ICA data extracted from the EyeWorks™ Analyze software were cut in multiple blocks from the two LN load up to the eight LN load. Finally, means and standard error of the mean (SEM) of scaled ICA values for each LN load were calculated.

### Statistical Analysis

Descriptive statistics were performed for demographic, clinical and psychometric data using Wilcoxon rank sum test (W) or chi-square test (*χ*^2^). Normality was tested with the Shapiro-Wilk test. The effect size was assessed using Cohen’s *d* and interpreted by Cohen’s criteria (small = 0.20; medium = 0.50; large = 0.80; Cohen, 1992). Pearson’s correlation coefficient (*r*) was calculated for each group to examine relationships between mean ICAs and other variables including demographic, clinical, and cognitive test scores utilized in this study. A linear mixed model with fixed effects was employed to analyze cognitive workload changes as a result of cognitive demand for both groups. The main effects of group (PD vs. controls) and demand (LN load), and interaction effect of group by demand were analyzed. The repeated effect of LN loads was analyzed using a correlation compound-symmetry structure, which showed the best model fit. Pairwise comparisons were conducted with a Sidak correction for multiple comparisons. Missing ICA variables, especially for the six LN load, were imputed with the last ICA values in the previous LN load (last observation carried forward). A second linear mixed model was employed to investigate the pattern of cognitive workload as a result of cognitive demand in high performers (i.e., those who successfully repeated seven or eight sequences) and low performers (i.e., those successfully repeated only five or six sequences), both in the PD and the control groups. For all tests, significance level was set at *p* = 0.05. All statistical analyses were performed using IBM SPSS Statistics v23.

## Results

### Participant Characteristics

A total of 26 participants were included in this study: 16 non-demented participants with PD and 10 healthy controls. The controls were matched for age, education and cognitive status (MOCA). There were more men in the PD group compared to the control group (*p* = 0.03; Table 1). Although the PD group scored lower in California Verbal Learning Test-II (*p* = 0.04) and Wechsler Memory Scale-IV Logical Memory I (*p* = 0.01) compared with the control group, none of PD participants scored two standard deviations below than the healthy controls, which indicated no cognitive impairment according to the Movement Disorder Society criteria (Litvan et al., 2012). The median disease duration was 6 years. Fifteen patients with PD were in Hoehn & Yahr (ON) stage 2; one was 2.5. No significant correlations were found between LED and both psychometric test performances and pupillary responses. Other clinical characteristics are shown in Table 1.

### Mean Cognitive Workload in Letter-Number Sequencing Test

The mean ICAs in LNS for PD and control groups were 0.34 ± 0.10 and 0.30 ± 0.09 respectively (*p* = 0.23). The effect size estimation using mean ICAs of both groups found a medium effect size (Cohen’s *d* = 0.49). No significant correlations were found between demographic, clinical, psychometric test scores (Table 1) and cognitive workload (ICA), except for performance on the Tower of London test that strongly correlated with mean ICA in the PD group (*r* = −0.65; *p* = 0.01) and education that strongly correlated with mean ICA in the control group (r = −0.70; *p* = 0.02).

### Cognitive Workload and Cognitive Demand in Letter-Number Sequencing Test

In the PD group (*n* = 16), no participant was able to complete the whole task until the eight LN load: five participants completed at seven LN load, 10 participants completed at six LN load, and one participant completed at five LN load. In the healthy control group (*n* = 10), two participants completed at eight LN load, three participants completed at seven LN load, three participants completed at six LN load, and two participants completed at five LN load. Maximum LN loads were not significantly different between the two groups (*χ*^2^ = 5.51, *p* = 0.14). All but three participants (one PD and two healthy controls) were able to complete at six LN load. Therefore, ICA data between two LN loads and six LN loads were used in the analysis.

**Figure 2:**
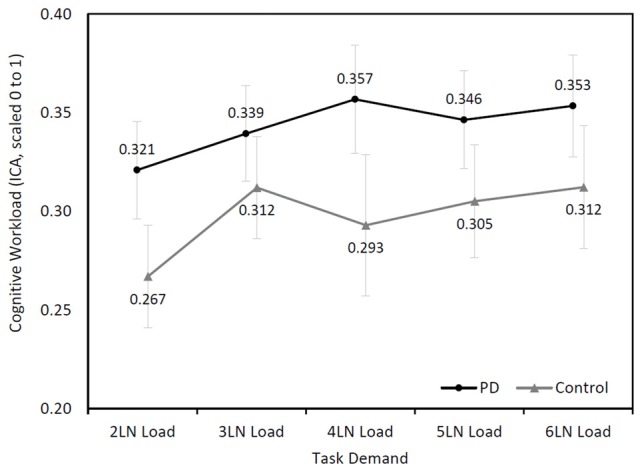
Mean (±SEM) changes of cognitive workload (Index of Cognitive Activity (ICA), scaled 0 to1) over cognitive demand during the letter-number sequencing (LNS) in the Parkinson’s disease (PD) and control groups.

The right eye ICA increased with increasing cognitive demand during the LNS in both groups (Figure 2). The mixed model analysis found a significant effect of cognitive demand on cognitive workload (*F*_(4,94.72)_ = 4.24, *p* = 0.003). Pairwise comparisons with a Sidak correction found significant differences between the LN loads 2 and 3 (*p* = 0.03), 2 and 4 (*p* = 0.04), 2 and 5 (*p* = 0.04), and 2 and 6 (*p* = 0.03).

Although participants with PD showed higher cognitive workload throughout the task, their ICA values were not higher than those of controls (*F*_(1,26.18)_ = 1.54, *p* = 0.23). No significant interaction effect was found between the two groups (*F*_(4,94.72)_ = 0.93, *p* = 0.45).

### Cognitive Workload and Maximum Letter-Number Load

**Figure 3:**
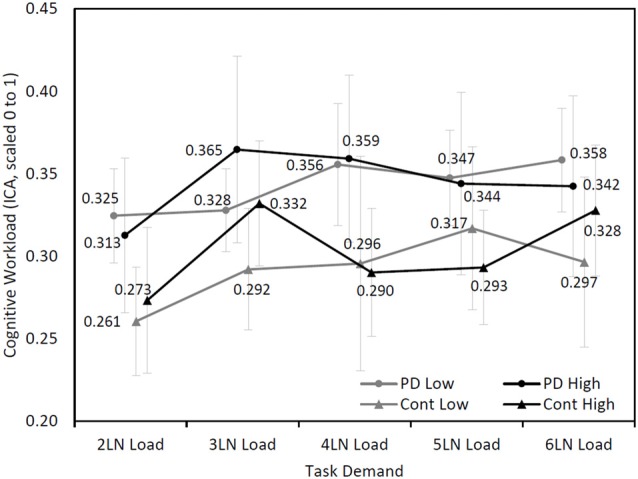
Mean (±SEM) changes of cognitive workload (ICA, scaled 0–1) over cognitive demand during the LNS between high performers (7 or 8 LN load) and low performers (5 or 6 LN load) in Parkinson’s disease (PD) and control groups.

Further analysis was performed based on the maximum LN load that participants completed. In the PD group, five participants completed seven or eight LN sequences (high performers) and 11 participants completed five or six LN sequences (low performers). In the control group, five participants were considered high performers and five participants were categorized as low performers. A mixed model analysis found an approaching significance in the interaction among cognitive demand, group (PD and control), and maximum LN load (high and low; *F*_(4,336.74)_ = 2.15, *p* = 0.07), while other main effects and interaction effects were not statistically significant (Figure 3). Further analysis showed that the high performers exhibited a different pattern of cognitive demand compared to low performers (*F*_(4,377.19)_ = 4.76, *p* < 0.001), regardless of disease status.

## Discussion

This study provides evidence that cognitive workload measured by pupillary response reflects cognitive demand in non-demented people with PD and in healthy controls. Both non-demented individuals with PD and healthy controls demonstrated the same pattern of cognitive workload with increased cognitive demand. The pattern of cognitive workload as a result of cognitive demand was different in high performers compared to low performers, regardless of disease status.

Recent studies suggested that pupillary response is an effective tool to investigate the amount of cognitive workload in people with PD (Wang et al., 2016; Orlosky et al., 2017; Ranchet et al., 2017b). Ranchet et al. (2017b) found a difference in pupillary responses between non-demented people with PD, demented people with PD, and healthy controls during a saccadic task. Ranchet et al. (2017b) used the ICA algorithm to calculate the amount of cognitive workload through pupil dilation whereas the rest of the similar studies (Wang et al., 2016; Orlosky et al., 2017) used pupil diameter change as a measure of cognitive workload. However, pupil size is mediated by changes in lighting, accommodation, and stress (McDougal and Gamlin, 2015). Even with perfect ambient conditions, there is still potential noise that may affect the accuracy of cognitive workload values. The ICA algorithm filters out these confounding factors (Marshall, 2007), and might therefore truly reflect pupillary response to cognitive activity. The novelty of the present study is that it demonstrated the responsiveness of pupillary response to increased levels of cognitive demand in people with PD and healthy controls. In the future, pupillary response might be used as a diagnostic tool to discriminate between non-demented people with PD and demented people with PD.

In the present study, both individuals with PD and healthy controls who completed the lower LN load displayed increased cognitive workload compared with individuals who completed higher LN load. The findings suggest that people who performed highly on the working memory task adopted a different cognitive workload pattern compared with low performers. At three LN load, high performing participants showed a steep increase in cognitive workload compared to the two LN load. It is possible that the high performers exhibited greater cognitive workload from two to three LN load since they were forming a strategy to tackle the task. After this strategy was formed, their cognitive workload decreased followed by a steady increase in cognitive workload with increased cognitive demand. The low performers, instead showed a steady increase in cognitive workload as a result of cognitive demand. This early increase in cognitive workload followed by a decrease has also been observed by Attar et al. (2016) during a working memory task. Our results therefore support the assumption that pupillary response is a valid measure of cognitive demand in both non-demented individuals with PD and healthy controls.

The mechanism of the pupillary response to cognitive workload is mediated by a combination of parasympathetic and sympathetic activity (Beatty, 1982; Sirois and Brisson, 2014). Increased activation of the locus coeruleus subsequently sends inhibitory signals to the Edinger-Westphal nucleus, leading to inhibition of pupil constriction muscles through parasympathetic fibers, resulting in pupil dilation (Beatty and Lucero-Wagoner, 2000). Also, increased activation of sympathetic fibers leads to activation of dilator pupillae, which results in pupil dilation (Sirois and Brisson, 2014). However, PD pathophysiology affects the autonomic nervous system and may lead to physiological differences in the pupil size compared to the healthy adults (Micieli et al., 2003). The ICA algorithm we used for cognitive workload analysis, filters out these physiological differences and calculates the change in pupil dilation resulting from increased cognitive workload (Marshall, 2007). Also, the correlation analysis showed no influence of autonomic dysfunction in ICA. Therefore, it is conceivable that our results were not affected by PD pathophysiology, and accurately reflects the amount of cognitive workload during incremental cognitive demand.

There are several limitations of this study. Our sample size was small; however, there was a medium effect size in mean cognitive workload difference between patients and controls. Therefore, the present study provides pilot results to calculate adequate power for future studies to better understand the cognitive workload differences during cognitive testing between non-demented people with PD, demented people with PD and healthy controls. Secondly, the two groups were not matched for sex. However, no significant correlations were found between cognitive workload and sex in either group.

## Conclusion

Cognitive workload measured by pupillary response is able to detect cognitive demand in non-demented people with PD and healthy controls. Pupillary response is a valid measure to cognitive demand in both non-demented individuals with PD and healthy controls. Pupillary response has properties of low cost, easy implementation, and low participant burden (Elman et al., 2017). In clinics, pupillary response to cognitive demand might be used to discriminate non-demented people with PD from demented people with PD. Future studies are warranted to confirm our findings with different cognitive tasks and to verify the use of pupillary response as a diagnostic tool for cognitive impairment in people with PD.

## Author Contributions

HD: conceptualizing the study. MK, SM and HD: drafting the manuscript and data analysis. HD, AEA, KEL and RP: valuable suggestions and manuscript review.

## Conflict of Interest Statement

The authors declare that the research was conducted in the absence of any commercial or financial relationships that could be construed as a potential conflict of interest.
